# Morphological Assessment of Concomitant Lesions Detected in Goat Herds Naturally Infected with Paratuberculosis (Johne’s Disease)

**DOI:** 10.3390/ani13101693

**Published:** 2023-05-19

**Authors:** Elena Plamenova Stefanova, Óscar Quesada-Canales, Yania Paz-Sánchez, María José Caballero, María del Pino Quintana-Montesdeoca, Antonio Espinosa de los Monteros, Miguel Antonio Rivero, Ayoze Castro, Valentín Pérez, Marisa Andrada

**Affiliations:** 1 Veterinary Histology and Pathology, Institute of Animal Health and Food Safety (IUSA), Veterinary School, University of Las Palmas de Gran Canaria, 35413 Arucas, Gran Canaria, Spain; elena.plamenova101@alu.ulpgc.es (E.P.S.); yania.paz102@gmail.com (Y.P.-S.); antonio.espinosa@ulpgc.es (A.E.d.l.M.); miguel.rivero@ulpgc.es (M.A.R.); ayoze.castro@ulpgc.es (A.C.); marisaana.andrada@ulpgc.es (M.A.); 2 Departament of Morphology, Veterinary School, University of Las Palmas de Gran Canaria, 35413 Arucas, Gran Canaria, Spain; 3 Departament of Mathematics, University of Las Palmas de Gran Canaria, 35017 Las Palmas de Gran Canaria, Gran Canaria, Spain; mariadelpino.quintana@ulpgc.es; 4 Departamento de Sanidad Animal, Facultad de Veterinaria, Campus de Vegazana, Universidad de León, 24007 León, Spain; valentin.perez@unileon.es; 5 Departamento de Sanidad Animal, Instituto de Ganadería de Montaña (CSIC-ULE), 24346 Grulleros, Spain

**Keywords:** *Mycobacterium avium* subspecies *paratuberculosis*, goat pathology, histopathology, lesions, vaccination, concomitant pathologies

## Abstract

**Simple Summary:**

Paratuberculosis (PTB) is a well-known disease with considerable financial impact on the farm industry worldwide. Nevertheless, data regarding the assessment of naturally infected goat herds is limited. The present study describes in detail the observed gross and histological lesions detected in 39 necropsies of goats (15 vaccinated and 24 non-vaccinated) from herds with a confirmed history of PTB. PTB microscopic lesions of various grades were detected in all animals in target organs and the presence of the causative agent was confirmed using different laboratory tools. The main inflammatory findings affected the hemolymphatic, respiratory and gastrointestinal systems. The lesions were confirmed microscopically with lesser macroscopically visible alterations. Our result demonstrated that non-vaccinated animals presented more severe PTB intestinal lesions and had respiratory inflammation in all age groups studied. Those also presented a higher prevalence of ileocecal valve PTB lesions. Gastrointestinal non-PTB lesions were detected in higher number in non-vaccinated goats. Thus, histology is a powerful tool for herd diagnosis and assessment. Mainly inflammatory lesions of the respiratory and gastrointestinal tract were detected in the studied PTB-affected herds. Additionally, vaccination against PTB could play a key role in the reduction of lung and gastrointestinal inflammatory processes present in the herd.

**Abstract:**

Paratuberculosis (PTB), caused by *Mycobacterium avium* subspecies *paratuberculosis* (MAP), causes significant financial losses in the ruminant industry. The aim of this study is to describe the concomitant pathological findings as well as PTB-induced lesions in 39 naturally infected goats (15 vaccinated and 24 non-vaccinated). All animals exhibited MAP-induced microscopic lesions affecting target organs, although only 62% of those were detected grossly. Mainly concomitant inflammatory pathologies were recognized affecting the hemolymphatic, respiratory and gastrointestinal systems. Non-vaccinated animals exhibited both moderate and marked granulomatous enteritis in contrast with vaccinated ones which presented mild intestinal affection. Our results demonstrate that non-vaccinated animals presented pneumonia in all age groups studied (from 12 up to >48 months old). A significantly higher prevalence of ileocecal valve PTB lesions was detected in non-vaccinated animals with pneumonic lesions (*p* = 0.027). Furthermore, a reduction of gastrointestinal non-PTB processes was described in vaccinated goats. In conclusion, a PTB infected goat herd can be affected by a wide range of concomitant pathologies, mostly inflammatory in origin. Anatomic pathology is of crucial importance for correct herd diagnosis and histopathology is an indispensable tool for lesion detection. Additionally, anti-MAP vaccination could have a beneficial effect on the reduction of respiratory and gastrointestinal non-PTB diseases.

## 1. Introduction

Paratuberculosis (PTB), also known as Johne’s disease, is a progressive emaciating disease caused by *Mycobacterium avium* subspecies *paratuberculosis* (MAP), which affects domestic and wild ruminants worldwide [1,2,3,4,5,6]. Although young animals are more susceptible to infection due to the high degree of environmental contamination, the subclinical phase is long, and the originated economic losses are significant in both cattle and the small ruminant industry [4,7,8,9]. Clinical-phase signs are non-specific and include a reduction in daily milk yield, weight loss, poor body condition and diarrhea [10].

PTB is a disease which is impacting European countries due to the considerable economic losses it originates and its high prevalence. Herd seropositivity of up to 65% has been reported in goat and sheep herds in Germany and 62.9% in France [11,12]. In Italy, various reports highlight the need for the implementation of control strategies and viable diagnostic tools for PTB early detection [13,14]. In Spain, goat farming is one of the biggest pillars of the national livestock breeding industry since the country counts for 23% of EU’s goat population (EUROSTAT 2021). Goat farms represent the primary livestock breeding sector in the Canary Islands, with 202.887 heads and 1289 dairy farms distributed as follows: 478 on Gran Canaria, 224 on Tenerife, 212 on Fuerteventura, 129 on La Palma, 117 on Lanzarote, 82 on El Hierro and 47 on La Gomera (ISTAC 2020). The archipelago has the fourth-largest goat population in Spain (Eurostat 2020), composed mainly of certified autochthonous endangered breeds (Orden APM/26/2018). In continental Spain, considerable economic losses due to goat PTB have been reported, and it is considered a widespread problem for the whole national territory with herd seroprevalence of 87.5% in southern Spain, 46% in Huelva region, 44% in Madrid and 56% in Avila, although data about the prevalence of the disease in the Canary Islands is limited [8,15].

Gross lesions in animals with PTB observed during necropsy are usually confined to the intestinal mucosa and the draining lymph nodes [6,16,17]. The ileocaecal valve mucosa can appear diffusely thickened and folded into transverse rugae, the crests of which may be congested. The mesenteric (MS) and ileocecal lymph (IC) nodes (LNs) are diffusely enlarged, edematous and pale (lymphadenomegaly, LAM). Lymphangitis can be detected in the mesentery. In some mild cases, lymphangitis is the only gross lesion specific enough to justify a presumptive PTB gross diagnosis [6,16,18]. Other commonly associated macroscopic findings are loss of muscle mass, serous atrophy of fat depots, intermandibular oedema, fluid effusion in cavities, with the presence of mineralization plates and intimal fibrosis in the thoracic aorta [6,16,18].

The main histopathological lesions associated with MAP-infection include transmural granulomatous enteritis, mesenteric and ileocecal lymph nodes granulomatous lymphadenitis and lymphangitis [6,16,17,18]. Villi can present moderate to marked atrophy with infiltrate of epithelioid macrophages and a variable number of Langhans-type multinucleated giant cells focally or diffusely distributed along the lamina propria, submucosa muscular layer or serosa of the intestine. The lesions observed in lymph nodes can vary from multiple foci of epithelioid macrophages up to well-formed encapsulated granulomas with central necrosis [6,16,17,18].

Although PTB is a major livestock farming issue of worldwide distribution, there may be a variety of other different diseases co-existing in naturally infected herds. The prevalence of the different diseases varies among the age groups present in the herd, the breed of the animals, the herd size, environmental factors as well as the variations in the pathogens present in each farm [8,15,19,20,21]. Respiratory, gastrointestinal, lymphatic and hepatic pathologies stand out among the main lesions detected in small ruminant herds worldwide [19,22,23].

Respiratory diseases represent 5.6% of the most common diseases in small ruminants affecting both individuals or groups, resulting in considerable financial losses due to decreased meat, milk and wool production along with a reduced number of offspring [24,25]. The etiology is multifactorial, involving mainly bacteria and viruses, and secondary infections are common. The lung can present lesions varying from acute bronchopneumonia up to severe chronic lung involvement [5,24,25].

Gastrointestinal affection, besides PTB-induced lesions, can be associated with diarrhea and weight loss due to viral, bacterial and parasitic infections. Acute lesions can vary from marked hyperemia of the intestinal mucosa up to fibrino-haemorrhagic and even necrotizing enteritis affecting both the small and large intestines. Subacute and chronic cases can exhibit multifocally distributed lesions, such as abscesses affecting the ileum, caecum and colon [6,19,22,23].

Lymphadenopathy is also considered a major clinical finding in many important infectious goat diseases. LNs are the primary disease location in cases of *C. pseudotuberculosis* induced caseous lymphadenitis [5,26]. Acute lymphadenitis, on the other hand, is a common finding in regional LNs associated with organs affected by bacterial infection [5,19,26].

Specific liver lesions are mainly of inflammatory origin (hepatitis) [5]. In adult animals, abscesses are commonly observed in association with *Trueperella pyogenes* or *Fusobacterium necrophorum* infection. Necrotizing hepatitis caused by *Clostridium* spp. is also a common finding [5,27,28].

Anatomopathological examination (gross and histopathological findings) is crucial to adequate herd diagnosis [16,18,29]. For correct assessment and confirmation of the suspected diagnosis or new insights on the investigated disease problem in the herd, all possible necropsies following a systematic procedure should be performed as a useful adjunct to clinical examination [5,19,22,25].

The present study aims to provide a morphological classification of a variety of MAP-induced lesions in naturally infected animals as well as to recognize the different concomitant pathological findings in 39 female majorera goats with histopathological PTB lesions detected during a routine postmortem examination.

## 2. Materials and Methods

### 2.1. Study Design

A total of 39 adult goats with histological PTB lesions were included in the study. All animals were submitted with a presumption of a field PTB diagnosis and were examined by the Anatomic Pathology Diagnostic Service of the Veterinary School of the University of Las Palmas de Gran Canaria. The animals were submitted from 8 farms (F1, F2, F3, F4, F5, F6, F7 and F8) in the Canary Islands with a confirmed history of PTB. The on-farm confirmation protocol included serological ELISA immunoassay (PARACHEK^®^ 2 Kit, Thermo Fisher Scientific, Waltham, MA, USA), bacteriological culture of environmental samples and subsequent PCR confirmation, postmortem examination and compatible clinical signs detection. A total of 15/39 animals from 3 of the farms were vaccinated against MAP (Table 1). All goats selected presented histological lesions compatible with PTB including granulomatous enteritis (GE) with the presence of focal to multifocal/diffuse transmural accumulations of epithelioid cells and lymphocytes typically accompanied by Langhans-type multinucleated giant cells (MGCs) and/or granulomatous lymphadenitis (GLA) of the mesenteric lymph nodes (MS LNs) characterized by the infiltration of epithelioid macrophages and/or Langhans-type MGCs.

According to the local legislation (Decree 51/2018 del 23 de abril), caprine vaccination against MAP is only permitted after “tuberculosis-free” status confirmation and PTB diagnostic confirmation since the archipelago was declared “officially free” of bovine tuberculosis (TB) in 2017. The vaccination protocols in the 8 farms studied included commercial vaccines against *E. coli*, *Clostridium* spp. (enterotoxemia), *Chlamydia* spp. (abortion) and *Pasteurella* spp. (bronchopneumonia) which were applied following the manufacturer’s recommendations.

### 2.2. Anti-MAP Vaccine

Gudair^®^ is a commercial heat-inactivated vaccine containing 2.5 mg/mL of MAP strain 316 F with mineral oil adjuvant (CZ Vaccines S.A., O Porriño, Pontevedra, Spain) for use in sheep and goats. It had been administered once subcutaneously as per the manufacturer’s instructions in the three farms applying PTB vaccination enrolled in this study.

### 2.3. Sample Collection and Processing for Histolgical Examination

Necropsies were performed following the protocol proposed by King et al., 2014 [29]. Lymph nodes and intestines were sampled for histology according to the recommendations of the national bovine tuberculosis eradication program protocol 2021 (MAPA, 2021) as follows: mesenteric (MS), ileocecal (IC), retropharyngeal (RPh), prescapular (PE) and mediastinal (MD) lymph nodes (LNs) and ileocecal valve region (ICV). Samples from representative organs (lungs and trachea, liver, kidneys and urinary bladder, heart, LNs, spleen, intestine, forestomaches and abomasum, uterus and ovaries, mammary gland, skeletal muscle and brain) were systematically collected for histological examination.

All tissue samples were fixed in 10% buffered formalin, embedded in paraffin and processed routinely, sectioned at 5 μm and stained with hematoxylin/eosin (HE). Additionally, selected tissue sections of 4 μm were stained with the Ziehl–Neelsen (ZN) technique for detecting acid-fast bacilli (AFB).

### 2.4. Gross Evaluation

All macroscopic lesions were recorded and described in terms of location, color, size, shape, consistency and number or percent of involvement of the affected organ and a presumed morphological gross diagnosis was established by an experienced pathologist.

### 2.5. Histological Evaluation

All histopathological findings were detailed and organized according to the system affected.

Two grading systems were applied to evaluate the PTB-compatible histological lesions. MS LNs lesions were classified as 0 (no lesions), I (initial), II (solid), III (minimal necrosis) and IV (necrosis and mineralization) as per Wangoo et al., 2005, grading score described for tuberculosis lymph node granulomas [30]. The lesions observed in the lamina propria (LP) and the Peyer’s patches (PPs) of the ICV were graded separately in terms of severity (mild, moderate and marked) and distribution (focal, multifocal and diffuse) using the grading system proposed by Krüger et al., 2015 [18]. Severity was graded as mild when only small circumscribed lymphocytic or granulomatous infiltrates were present with no change of tissue architecture; moderate when granulomatous infiltrates with altered tissue architecture were present; and marked when massive granulomatous infiltrates with partially or completely disrupted tissue architecture were observed [18]. Lesion distribution was considered focal when up to 3 distinct granulomatous infiltrates were observed per section; multifocal when more than 3 granulomatous distinct infiltrates per section were seen; and diffuse when granulomatous infiltrate was present throughout the whole section [18].

### 2.6. MAP Confirmation by Immunohistochemistry

Selected sections (3 μm) from MS LNs and ICV were immunohistochemically labelled with a polyclonal anti-MAP in-house antibody kindly provided by Dr. V. Perez, University of León, León, Spain. A polymer-based detection system (EnVision^®^System Labelled Polymer-HRP; Dako, Glostrup, Denmark) was employed, following the manufacturer’s instructions. Subsequently, immunolabelling was developed with a commercial solution of 3,3′diaminobenzidine (DAB) (K3468; Dako, Glostrup, Denmark). The slides were counterstained with Harris´ hematoxylin and mounted in a hydrophobic medium.

The number of mycobacteria per section was graded using a scoring system described by Krüger et al., 2015 [18], as follows: none (<2 labelled bacteria per section), single (mycobacteria in <20% epithelioid cells and/or MGCs; single/few bacteria per cell or foci of granular labelling, predominantly <21 mm in diameter), few (mycobacteria in 20% to 50% epithelioid cells and/or MGCs; on average, 1 to 10 bacteria per cell or foci of granular labeling predominantly >21 mm in diameter) and many (mycobacteria in >50% to 75% epithelioid cells and/or MGCs; on average >10 bacteria per cell, with up to 50% of cells containing countless bacteria).

### 2.7. qPCR and Bacterial Culture for MAP Identification

In order to confirm MAP infection, frozen tissue samples from MS LNs and ICV were submitted to external laboratories for real-time polymerase chain reaction (qPCR) and bacterial culture. Frozen tissue samples from 29/39 animals were available for examination. Molecular biology qPCR targeting IS900 gene was performed on samples 13/29 animals using EXOone qPCR kit (EXOPOL SL, Zaragoza, Spain). Samples were considered positive when Cq ≤ 38. Tissues from 16/29 animals were examined by bacterial culture as part of the national tuberculosis eradication and surveillance program. Samples from 3/29 animals were submitted to both qPCR and bacterial culture exams. The culture analysis was carried out by the national infectious diseases reference laboratory of VISAVET, Health Surveillance Centre, Madrid, Spain. The qPCR was performed by a private external laboratory.

### 2.8. Statistical Analysis

An observational cross-sectional study was carried out. The prevalence of macroscopic and microscopic pathological findings was calculated separately in each organic system affected. Lesion type prevalence was also measured in each system affected analyzing gross and histological findings separately.

Statistical analysis of data was performed by IBM SPSS Statistics 27 (IBM Corp. Released 2020. IBM SPSS Statistics for Windows, Version 27.0. Armonk, NY, USA: IBM Corp). Categorical variables were summarized using percentages and relative or absolute frequencies. The ages of the studied goats were categorized in the following groups: (0–12] months, (12–24] months, (24–36] months, (36–48] months and >48 months. Chi-square test was used to contrast the association between two categorical variables. To analyze the association between two ordinal scale variables, Kendall’s Tau-b test was applied. Numerical variables were summarized using the mean, standard deviation (SD), median and interquartile range (IQR). A Shapiro–Wilk test was applied to analyze the sample normality. A non-parametric Mann–Whitney U test was used to compare two independent samples when the condition for normality of the numerical variable was not met. Agreement between qualitative results obtained by two measurement procedures was calculated using the kappa coefficient (ĸ). The results were considered statistically significant if the *p* value < 0.05.

## 3. Results

### 3.1. Necropsy Findings

All animals studied exhibited poor body conditions characterized by severe emaciation, protrusion of lumbar vertebrae and easily palpable transverse processes, muscle mass loss and a reduction of visceral fat deposits.

Data about the age of the animals was available in 38/39 cases. The studied animals were between 7 and 121 months of age, with a mean of 29 months, median of 16.50 months, SD of 30.2 months and IQR of 16 months. The ages of the studied goats were categorized in the following groups: (0–12] months, (12–24] months, (24–36] months, (36–48] months and >48 months in which 1, 21, 10, 2 and 4 animals were included, respectively.

**Figure 1:**
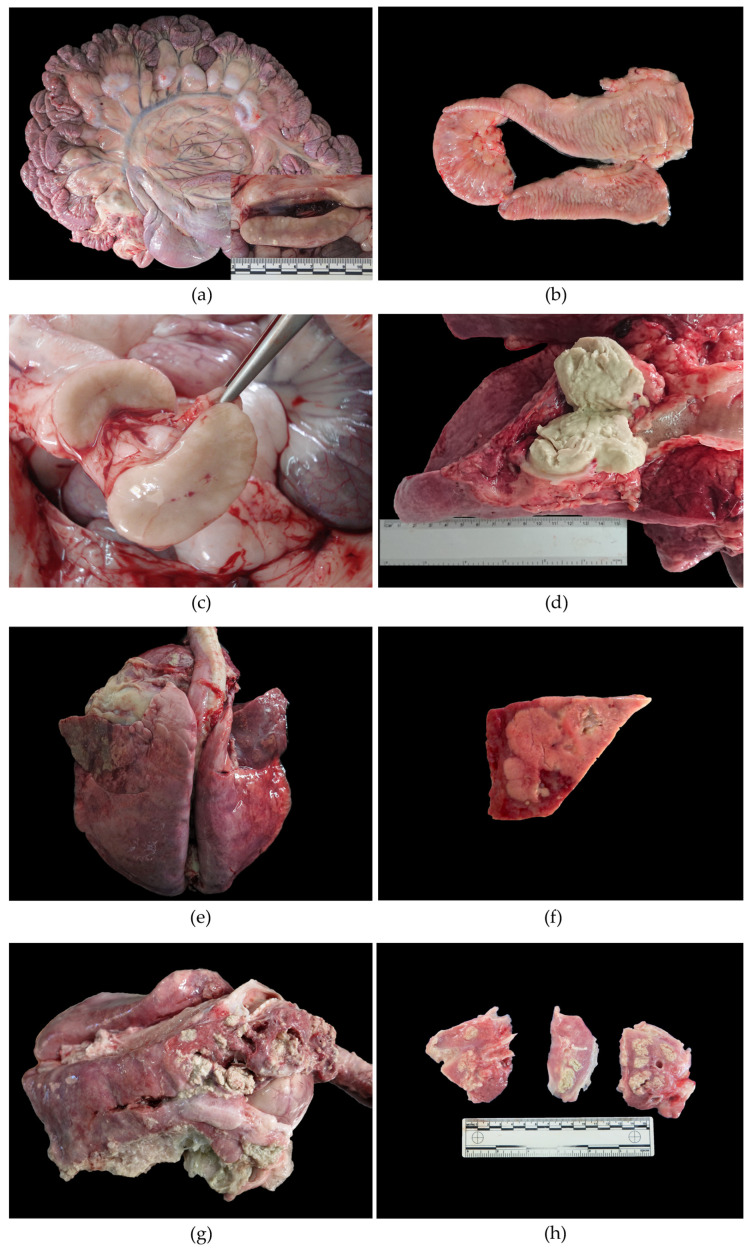
Main gross findings in 39 naturally infected goats with PTB lesions. (**a**) Severe lymphadenomegaly (LAM) affecting the mesenteric lymph nodes (MS LNs); inset: MS LNs with white calcified granulomas on the cut surface. (**b**) Jejunum. Diffuse thickening of the intestinal mucosa folded into transverse rugae, characteristic of PTB. (**c**) Lymphadenomegaly (LAM) in an MS LN. Enlarged lymph node with uniform cut surface. (**d**) Caseous lymphadenitis (CLA) affecting mediastinal lymph nodes (MD LNs) with abundant caseous material replacing the parenchyma completely. (**e**) Cranioventral bronchopneumonia (CBP) characterized by cranioventral dark red consolidation of lung lobes. (**f**) CBP lung section showing whitish areas of necrosis and purulent exudate. (**g**,**h**) Severe CBP affecting 70% of the lung with abundant purulent exudate oozing from airways.

PTB granulomatous lesions were observed in 61.5% (24/39) of the animals affecting the MS LNs (Figure 1a and Table 2). PTB intestinal gross lesions were described in 7/39 (17.9%) cases consisting in the thickened, corrugated mucosal surface of the small intestine and the ICV region (Figure 1b and Table 2).

A significant difference was observed between the month of age of the animals and the presence of macroscopic PTB granulomatous lesions affecting the MS LNs (*p* = 0.022). Once the ages of the vaccinated and non-vaccinated groups were compared in relation with PTB induced lesions in MS LNs, significant variation was observed only in the non- vaccinated group (*p* = 0.029) in contrast with the vaccinated where the animals were mostly between 12 and 24 months (*p* = 0.471).

Granulomatous lymphadenitis was also detected in the RPh (2/39; 5.1%) and the mammary (MA) (2/39; 5.1%) LNs. Those LNs appeared firm and enlarged, with a thickened capsule. The cut surface revealed the partial to complete loss of the architecture, replaced by multiple white, firm poorly demarcated areas which often presented central caseous necrosis and mineralization.

Lymph nodes were one of the most affected organs (80%; 31/39). Lymphadenomegaly was detected in 64% (25/39) of the animals affecting mainly the MS (21/25), IC (9/25) and MA (9/25) LNs (Figure 1c). Inflammatory lesions (lymphadenitis) were seen in 57% (22/39), affecting different LNs.

Suppurative lymphadenitis (SLA) was observed in the PE (6/39; 15.4%) and MD (3/39; 7.7%) LNs. Those LNs appeared enlarged, soft and hyperemic with thick fibrous well-formed capsules. On the cut surface, the parenchyma bulged and exudated variable amounts of blood, pus or lymph.

Caseous lymphadenitis (CLA) characterized grossly by the classic “cheesy gland” appearance parenchyma affected the RPh (2/39; 5.1%), MD (3/39; 7.7%) and MA (2/39; 5.1%) LNs. Those LNs were large and firm, exudating variable amounts of pasty yellowish material (pus) which completely substituted the organ’s parenchyma (Figure 1d).

Abscess formation characterized by the complete substitution of the normal lymph node parenchyma by large amount of yellow-green pus surrounded by a thick fibrous capsule was seen affecting the RPh (3/39; 7.7%), PE (4/39; 10.3%), MD (2/39; 5.1%) and MA (1/39; 2.6%) LNs.

The respiratory tract was affected in 17/39 (43.6%), mainly by bronchopneumonia (CBP) (16/39; 41%) characterized by cranioventral consolidation of the lungs, affecting at least 30% of the parenchyma, with dark red to maroon discoloration and an oedematous cut surface (Figure 1e,f). In some cases, purulent or catarrhal material oozed from the small airways. Fibrous adhesions between the parietal and visceral pleura (fibrous pleuritis) were observed in 5/39 (12.8%), along with CBP. The presence of fibrin strands or vails on the pleural surface (fibrinous pleuritis) along with CBP was described in seven (17.9%) (Figure 1g,h). No association with PTB macroscopic lesions was observed (*p* = 0.792) and no differences between the ages of the animals affected were demonstrated (*p* = 0.854).

The gastrointestinal tract exhibited gross lesions not categorized as PTB, in particular, a nonspecific diffusely thickened appearance in 10/39 animals (25.6%) and hemorrhagic enteritis in one animal (2.6%). The lesions detected had statistically significant associations with PTB gross lesions in MS LNs only in the non-vaccinated group (*p* = 0.013).

The liver exhibited a uniform pale yellow color, round edges and friable texture (steatosis) in 7/39 (17.9%) of the cases and multifocal, white, firm, well demarcated areas of capsular and/or subcapsular fibrosis in 6/39 (15.4%). One animal presented multifocal well-demarcated mineralization.

The heart was affected in 13% (5/39) of the cases by a variety of lesions including hydropericardium (2/39; 5.1%), fibrous pericarditis (2/39; 5.1%) and petechial haemorrhages on the pericardial surface (2/39; 5.1%).

Kidneys were affected in 5% (2/39) of the cases, presenting multiple small white nodules of up to 1 cm diameter throughout the cortex, consistent with nonsuppurative interstitial nephritis.

A summary of the main gross findings affecting the 39 goats included in the study can be found as Appendix A (Table A1).

### 3.2. Microscopical Findings

**Figure 2:**
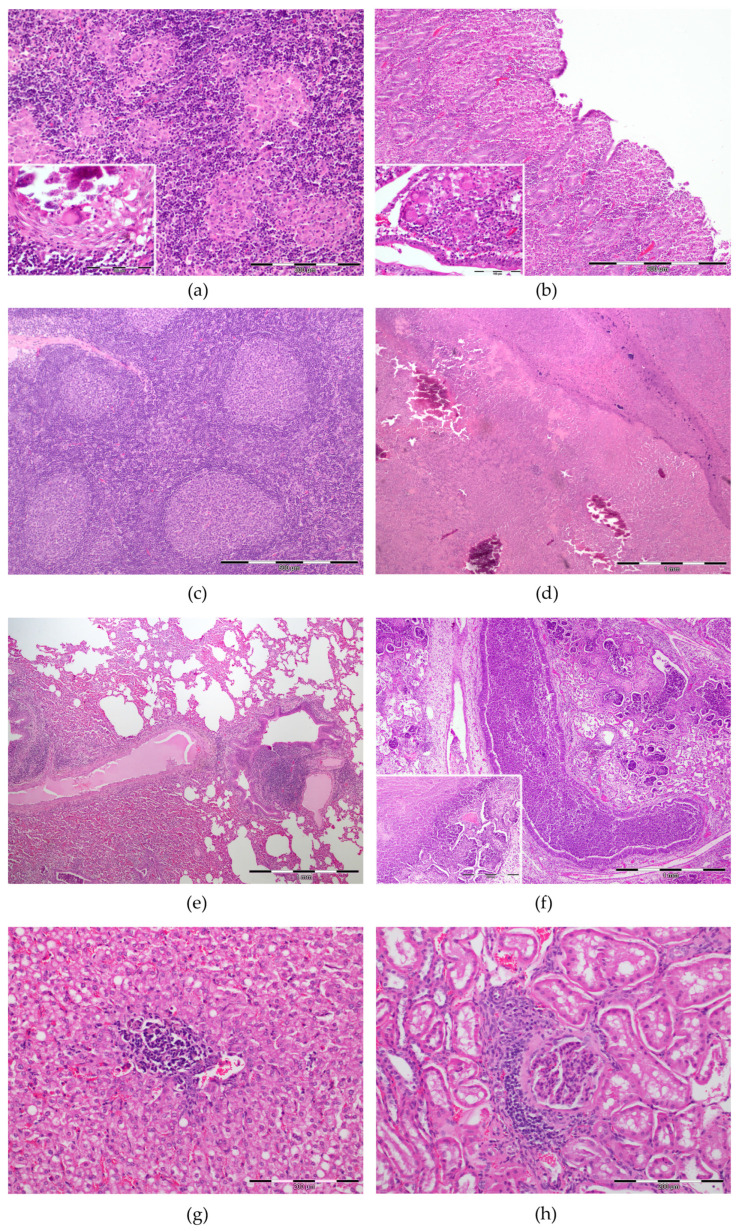
Main histopathological findings in 39 naturally infected goats with PTB lesions. (**a**) Mesenteric lymph node (MS LN), scale: 200 µm: multifocal aggregates of epithelioid macrophages inset (scale: 100 µm): Langhans-type multinucleated giant cells (MGCs) in a calcified granuloma. (**b**) Jejunum (scale: 500 µm): granulomatous enteritis formed by groups of macrophages and diffuse infiltration of lymphocytes; inset (scale: 100 µm): Langhans-type MGCs with epithelioid macrophages aggregates in intestinal villi tip. (**c**) Lymph node (LN) (scale: 500 µm): reactive lymphoid hyperplasia characterized by large lymphoid follicles with prominent germinal centers. (**d**) LN (scale: 1 mm): caseous lymphadenitis/suppurative lymphadenitis (CLA/SLA) with vast areas of lytic necrosis with mineralization. (**e**) Lung (scale: 1 mm): bronchointerstitial pneumonia (BIP) characterized by proliferation of type II pneumocytes, associated lymphoid tissue and thickening of the alveolar septa by lymphoid infiltration. (**f**) Lung (scale: 1 mm): severe fibrinonecrotizing bronchopneumonia (FNBP) with degenerated neutrophils, fibrin and necrotic debris filling bronchioles and alveoli and large areas of coagulative necrosis (inset, scale: 500 µm). (**g**) Liver (scale: 200 µm): perivascular lymphohistiocytic hepatitis characterized by foci of lymphocytes and occasional macrophages and mild steatosis. (**h**) Kidney, cortex (scale: 100 µm): interstitial nephritis with lymphohistiocytic infiltration expanding the interstitium.

Lesions consistent with PTB were observed in 100% of the cases (granulomatous enteritis and/or granulomatous lymphadenitis of the MS LNs). In affected MS LNs (92.3%, 36/39), grade IV granulomas were described in 27/36 (75%) of the animals (Figure 2). Although, severe granuloma formation (grade IV) was not always the main PTB lesions detected in affected LNs. In fact, in 16/36 (44%) of the cases, grade I multifocal microgranulomas constituted the predominant lesion type in the examined samples (Figure 2a). All vaccinated goats (15/15, 100%) exhibited grade IV lesions in contrast with the non-vaccinated ones where 7/24 (29.2%) and 2/24 (8.3%) animals only presented grade I and grade III lesions, respectively (Table 2). The PPs could be evaluated in 33 animals, 70% (23/33) of which presented PTB-compatible lesions of mild severity (46%, 15/33) and multifocal distribution (55%, 18/33). The LP was affected in 51.3% (20/39) of the cases, mainly by mild lesions (35.9%, 14/39) with multifocal distribution (48.7%,19/39) (Figure 2b and Table 2). In the vaccinated group, 9/15 (60%) animals were affected, presenting mild ICV lesions affecting the LP. In contrast, the non-vaccinated PTB affected goats had moderate and marked lesions in 4/24 (16.7%) and 2/24 (8.3%) of the cases, respectively. No significant differences were described between the age groups of the animals studied (*p* = 0.085), as well as between the medians of the vaccinated (14 months) and non-vaccinated (17 months) goats (*p* = 0.691).

Reactive hyperplastic lesions consisted in follicular hyperplasia with larger follicles with a paler germinal center containing an increased numbers of apoptotic lymphocytes and tingible body macrophages were observed in 53% of the animals affecting different lymph nodes (Figure 2c).

LNs were mainly affected by CLA and SLA (Figure 2d). CLA was characterized by variable-sized areas of lytic necrosis admixed with degenerated and viable neutrophils surrounded by epithelioid macrophages, lymphocytes and plasma cells, and a thick capsule of fibrous connective tissue. On the other hand, SLA LNs exhibited marked hyperaemia with diffuse infiltration of a moderate to large number of neutrophils, vast areas of necrosis and mineralization. The affected lymph nodes were the RPh, PE, MD and MA with lesions observed in 8/39 (20.5%), 4/39 (10.3%), 8/39(20.5%) and 4/39 (10.3%) of the cases, respectively. Abscess formation was confirmed only grossly and was not included in the histological analysis. The GLA described grossly in RPh and MA LNs were histologically classified as SLA/CLA.

Various organs also showed diverse and different lesions, mainly the respiratory tract, digestive tract, liver, cardiovascular system and kidneys.

The main lesions observed in the respiratory tract were inflammatory pneumonia processes (64.1%, 25/39) (Table 2). Necrotic-suppurative bronchopneumonia characterized by the presence of viable and degenerated neutrophils, fibrin and cell debris, filling bronchioles and alveoli, admixed with areas of necrosis and haemorrhage was detected in 12/39 animals (30.8%) (Figure 2f). Lymphohistiocytic interstitial pneumonia (IP) with increased number of lymphocytes and histiocytes thickening the alveolar septa was diagnosed in 6/39 (15.4%) of the cases. Bronchointerstitial pneumonia (BIP) was described in 3/39 animals (7.7%) and included bronchiolar necrosis, diffuse alveolar damage, thickening of the alveolar septa by lymphoid infiltration and hyperplasia of type II pneumocytes, perivascular lymphoid infiltration and hyperplasia of associated lymphoid tissue (Figure 2e). In 4/39 cases (10.3%), the lungs presented only fibrous pleuritis. In the vaccinated and non-vaccinated animals, pneumonia lesions were detected in 8/15 (53.3%) and 17/24 (70.8%), respectively (Table 2).

**Figure 3:**
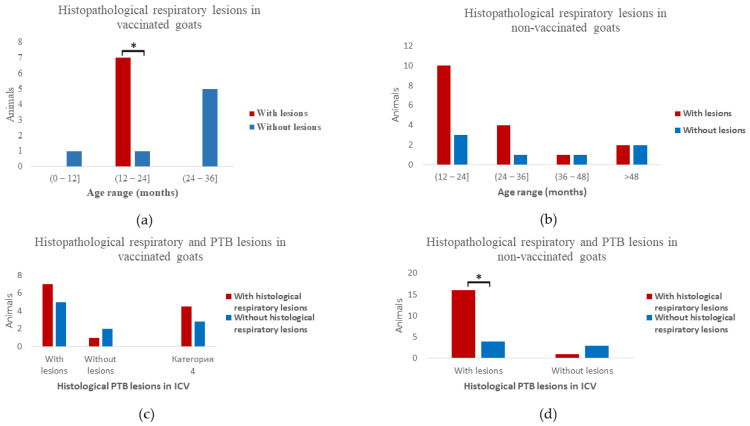
Associations between age groups, histopathological respiratory lesions and PTB histological lesions affecting the ileocecal valve (ICV) in vaccinated and non-vaccinated goats. (**a**) Distribution of vaccinated goats in different age groups in relation with presence of histopathological lesions of the respiratory tract. * *p* = 0.035 animals affected were mainly >12 < 24 months of age. (**b**) Distribution of non-vaccinated goats in different age groups in relation with presence of histopathological lesions of the respiratory tract. (**c**) Associations between the presence of histopathological respiratory lesions and histopathological PTB lesions in the ICV of vaccinated goats. (**d**) Associations between the presence of histopathological respiratory lesions and histopathological PTB lesions in the ICV of non-vaccinated goats. * *p* = 0.027 more non-vaccinated animals with PTB lesions affecting the ICV presented histological respiratory lesions.

Differences between the age groups of vaccinated and non-vaccinated animals were detected regarding the presence of microscopic lung inflammation. Most of the vaccinated animals with histological lesions had between 12 and 24 months of age (*p* = 0.035) (Figure 3a). Those lesions in the non-vaccinated goats were distributed in ages from 12 months up to >120 months, with no significant differences between the number of animals in each age range (*p* = 0.677) (Figure 3b).

In relation to the presence of histological inflammation affecting the respiratory tract (25/39), grade IV granulomas in the MS LNs were present in 16/25 (64%) animals (*p* = 0.145). Furthermore, significantly more non-vaccinated animals with MAP-induced lesions in the ICV presented lung inflammation (*p* = 0.027) (Figure 3d). No such association was described in the vaccinated group (*p* = 0.438) (Figure 3c).

Regarding the digestive system, lymphoplasmacytic (LPE) and eosinophilic enteritis (EE) were the main findings observed. LPE, characterized by a diffuse discrete-moderate number of lymphocytic mucosal infiltrate, was detected in 1/39 (2.6%) of the animals. EE characterized by eosinophils with a smaller number of globular leucocytes and plasma cells, infiltrating and expanding the intestinal mucosa was described in 2/39 (5.1%). The presence of coccidia of *Eimeria* spp. was confirmed in one animal with EE. Mild lymphohistiocytic abomasitis or gastric non-perforated ulcers were described in 4/39 (10.3%) of the animals.

There was no difference between the ages of the eight animals affected (*p* = 0.430). Seven (7) of those were non-vaccinated and only one was vaccinated (*p* = 0.090). Five (5) of the non-vaccinated affected goats (71%) exhibited grade IV granulomas affecting the MS LNs (*p* = 0.170).

The most frequent hepatic finding was lymphohistiocytic hepatitis (64.1%, 25/39), characterized by multiple foci of lymphocytes and occasional macrophages, mainly with a periportal distribution (Figure 2g). Hepatocellular steatosis with hepatocytes presenting one large or multiple variably sized discrete intracytoplasmic vacuoles, frequently displacing and compressing the nucleus to the periphery, was also observed (11/39, 28.2%).

Kidneys presented non-suppurative interstitial nephritis, mostly affecting the cortex and to a lesser extent, the medulla, with the diffuse infiltration of lymphocytes and occasional plasma cells and macrophages, interstitial fibrosis and tubular atrophy in 25.6% (10/39) of the cases (Figure 2h).

The heart was predominantly affected by the presence of apicomplexan schizont, containing large numbers of oval basophilic bradyzoites, compatible with *Sarcocystis* spp., expanding cardiomyocytes (9/39, 23.1%). Non-suppurative myocarditis characterized by multifocal lymphocytic infiltration and occasional myofiber degeneration was also observed (3/39, 7.7%). Fibrous pericarditis was confirmed in 3/39 (7.7%).

A summary of the histological lesions detected in the 39 animals studied can be found as Appendix A (Table A1).

### 3.3. MAP Identification (Immunohistochemistry, Ziehl–Neelsen, Bacterial Culture and qPCR)

**Figure 4:**
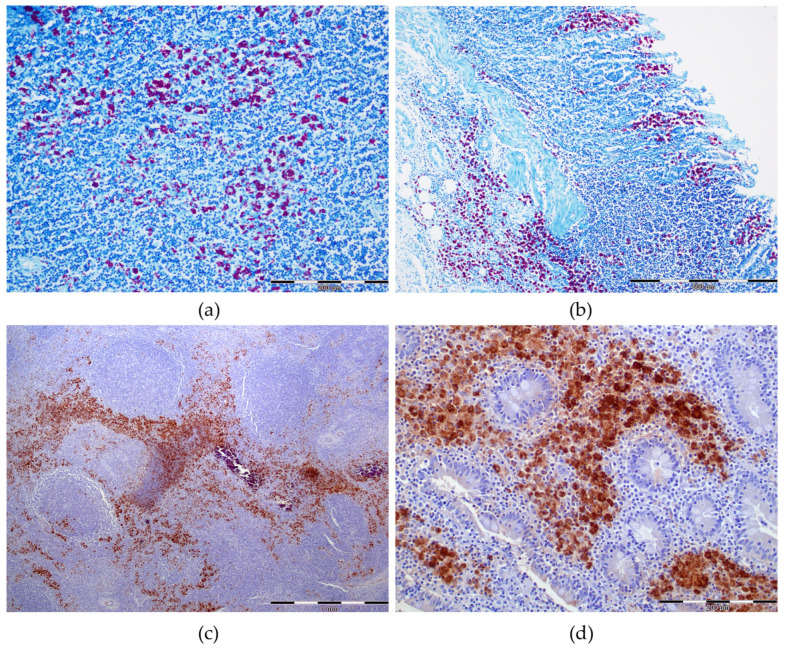
MAP confirmation by Ziehl–Neelsen stain (ZN) and immunohistochemistry (IHC) in mesenteric lymph nodes (MS LNs) and ileocecal valve (ICV). (**a**) MS LN, ZN (scale: 200 µm): numerous acid-fast bacilli (red) in the cytoplasm of macrophages and multinucleated giant cells; (**b**) ICV, ZN (scale: 500 µm): large number of acid-fast bacilli (red) in both lamina propria (LP) and Peyer’s patches (PPs) of severely affected ICV; (**c**) LN, IHC (scale: 1 mm): strong immunoreaction in an affected LN showed as brown granular cytoplasmatic stain in macrophages and multinucleated giant cells; (**d**) ICV, IHC (scale: 200 µm): strong brown granular immunolabelling in ICV mucosa.

In all cases included in the study, MAP positivity was confirmed in MS LNs and/or VIC by at least one of the techniques used. Positivity by both IHC and ZN was detected as cytoplasmic infiltrate in epithelioid macrophages and giant cells. In the case of IHC, immunoreaction was seen as brown cytoplasmic granules and as red bacilli by ZN (Figure 4). In MS LNs, results for ZN and IHC staining suggested MAP positivity in 82.1% (32/39) and 84.6% (33/39) of the cases, respectively (Table 3). The LP was positive by ZN in 53.9% (21/39) and in 59% (23/39) by IHC. The PPs were present in 33/39 of the cases and AFB were observed in 61% (20/33) by ZN and MAP-antigens were detected in 67% (22/33) by IHC. Moderate correlation between the detection capacity of ZN and IHC in MS LNs (κ = 0.537), ICV LP (κ =0.584) and ICV PP (κ = 0.609) was demonstrated. The main difference between the two techniques was observed in the intensity of the reaction which was stronger with IHC.

A total of 29/39 animals were examined for MAP confirmation by qPCR (13/29) and/or bacterial culture (16/29). Four animals (4/16, 25%) tested positive by bacterial culture and 9/13 (69.2%) by qPCR. Samples from 3/29 animals were tested with both techniques, and all were identified as negative by bacterial culture and as positive by qPCR. The same three animals were positive by both ZN and IHC.

## 4. Discussion

Paratuberculosis is an important health issue affecting the goat farm industry worldwide [4]. Complete postmortem analysis is crucial for adequate diagnosis and herd status confirmation [5,6,16,29]. Nevertheless, gross PTB-compatible lesions were only observed in 62% of the animals enrolled in this study and the majority were confined to the MS LNs. Similar results have been previously reported in both experimentally [18] and naturally [16] infected goats, highlighting the importance of the histopathological examination of the MAP target organs.

Regarding the vaccination status, vaccinated animals exhibited gross PTB lesions primarily between 12 and 24 months in contrast with non-vaccinated goats where granulomatous MS LNs lesions were detected macroscopically in all age groups including older animals. Our results agree with previous studies which confirm that the anti-MAP vaccination therapeutic effect consists in the reduction of the severity of lesions and clinical signs in both early vaccinated animals (around 5 months of age) and adult goats [2,10,21,31,32].

Granuloma formation is commonly reported but not always present in MS LNs of goats with PTB in both experimental [18] and natural infection [4,6,16,17]. Thus, an adapted score, proposed by Wangoo et al., 2005 [30], for tuberculosis-induced lesions was applied in the present study. Different scores for PTB histological evaluation in goats have been described, although none of them was selected since they are majorly centered in the PPs and LP lesions and do not grade the MS LNs granuloma stages separately [16,18].

Lesions observed in the MS LNs in our study were characterized by the presence of well-formed granulomas with vast areas of calcification in 24/39 cases, but in only 16/24 were both LAM and GLA described. To our knowledge, there is no data regarding the relation between gross LMA and GLA lesions [4,6,16,17]. Thus, the correct gross inspection of the cut surface is of crucial importance for the macroscopic diagnosis of PTB lesions, even in not apparently enlarged LNs.

MS LNs presented histologically detectable granulomatous lesions in 36/39 of the cases. In 27/36 of the animals, grade IV lesions were described, although in 16/36, grade I microgranulomas were the most frequent lesion type. Those results suggest that granuloma formation has a chronic evolution which has been confirmed in other mycobacterial infections such as cow tuberculosis [30,33,34]. Our findings also highlight that histological confirmation is needed for lesion detection in the early stage of granuloma formation.

Gross PTB lesions affecting the intestines were detected in only 7/39 of the cases. The absence of macroscopic lesions has been previously described as a common finding in goat PTB [3,4,16,17] in contrast with bovine PTB, where classic diffusely thickened corrugated intestinal mucosa folds are commonly observed [6,35]. Some authors describe ulceration of the intestinal mucosa in both clinical [36] and subclinical cases [18], but such findings were not observed in the animals studied. Histological evaluation of the intestine was carried out following the score proposed per Krüger et al., 2015 [18]. This score is based on histological lesions observed only with hematoxylin-eosin stain and is not influenced by AFB load detected with ZN as the one proposed by Corpa et al., 2000 [16], for naturally infected goats [16]. Modifications were introduced to separately evaluate the LP and the PP based on differences described in previous studies regarding the difference between the type of lesions described in those two locations [16,18]. Regarding the histological lesions described, our result agrees with previous studies which suggest that goats have limited ability to control mycobacterial infections and focal lesions are rarely seen [16,37].

MAP confirmation in formalin fixed-paraffin embedded tissues was carried out by ZN and IHC. Moderate correlation was observed between the detection capacity of the two techniques, but the intensity of the reaction was stronger in the case of IHC, agreeing with previous reports [38,39]. Low ZN staining could be explained by the fact that only the intact bacilli take up the stain and IHC anti-MAP antigens can detect dead bacterial cells with compromised cellular wall [38,39].

Etiological confirmation was also performed by qPCR/bacterial culture in 29/39 animals, 13 of which tested positive. Although bacterial culture is considered the gold standard for MAP detection, three animals which tested negative were positive by qPCR, IHC and ZN. Various studies describe qPCR as a more sensitive test for MAP detection in different species than bacterial culture, although data in goats is limited [35,40].

Both PTB and respiratory disease affect the goat farming industry worldwide, causing significant financial losses [12,17,30,31,32,33]. Nevertheless, data about the prevalence of both entities in goat herds is limited. In this study respiratory lesions were observed in 64.1% of the animals. Inflammatory lung disease was histologically detected more frequently in animals with PTB lesions in the ICV. Therefore, in naturally infected herds, there could be significant relations between the development of those two processes. Based on PTB pathogenesis and the demonstrated long subclinical stage and chronic onset [6,16,32,41], we could hypothesize that it has been the predisposal factor for lung inflammation in the present study. Additionally, vaccination against *Pasteurella* spp. was commonly issued as a control strategy in the farms studied and could have played a role, although, it is worth mentioning that lung disease in goats is multifactorial, including different causative agents and various environmental, management, herd and breed factors [24,25,42].

Pneumonia lesions had a 17.5% higher prevalence in the non-vaccinated animals in contrast with vaccinated ones. Furthermore, the majority of the vaccinated animals which exhibited histological lesions of pulmonary inflammation had between 12 and 24 months. In non-vaccinated animals, respiratory lesions were diagnosed histologically in different age groups including older animal. Additionally, significantly more non-vaccinated goats presented PTB lesions affecting the ICV, although no such difference was demonstrated regarding the MS LNs. Various studies have confirmed that vaccination against PTB has a generalized beneficial effect, reducing the overall number of losses in the herd, regardless of the cause, although there are no specific reports on reduction of respiratory inflammation [2,10,32,43].

Bronchopneumonia lesions could be associated with opportunistic bacterial pathogens including *Mannheimia haemolytica*, *Pasteurella multocida*, *Histophilus somni*, hemolytic *Streptococci*, *Helcococcus ovis* and *Mycoplasma* spp. [15,16,17,30]. Interstitial and bronchointerstitial pneumonia, on the other hand, have been described in a broad range of both infectious and non-infectious processes such as virus, mycoplasmosis, parasitosis, protozoal infection, toxicity and hypersensitivity reactions [19,24,25,42,44]. Since histological appearance is similar irrespective to etiology, diagnosis is usually based on clinical signs observed and lesions in other organs [15,16,17,30], and ancillary tests (e.g., PCR, microbial culture) are needed to identify the aetiologic agent [19,24,25,42,44].

Besides PTB, other lesions were described affecting the gastrointestinal tract including mainly lymphohistiocytic and eosinophilic enteritis which could be associated with parasitic agents such as *Ostertagia*, *Nematodirus* and *Trichostrongylus* [6,19,45].

In the present study, 15.3% more of the non-vaccinated animals presented non-PTB gastrointestinal lesions, although no statistical differences were demonstrated, probably due to the small sample size. Previous studies report a general reduction of the losses in vaccinated herds but do not analyze the different systems separately which were less affected [2,10,32,43]. Our results do suggest that anti-MAP vaccination could in fact have a protective effect against concomitant gastrointestinal pathologies.

Non-PTB lymphadenitis, on the other hand, was detected grossly in more than 50% of the animals. This reflects the invasion of infectious agents in the lymph node resulting from the drainage of products of a distant inflammation and their respective progression involving the node directly [26]. In the cases of *Corynebacterium pseudotuberculosis* compatible CLA, where LNs are the target organs, our results reported RPh, MD, and MA LNs as common lesion sites, which agrees with recent reports in goats [46,47].

The liver was mainly affected by randomly distributed multifocal lymphohistiocytic hepatitis. This unspecific inflammatory pattern is frequently reported in ruminants as secondary to systemic bacteremia caused by the agents affecting other organs [6,27]. Hepatocellular steatosis was also observed both grossly and histologically. Since all animals included presented PTB-attributed weight loss, the steatosis is most likely caused by the excessive entry of fatty acids into the liver due to their increased metabolization from the adipose tissue [6,27]. The granulomatous hepatitis recorded in only three animals could be associated with severe cases of PTB due to MAP spreading [4,6].

The cardiovascular system presented gross lesions only affecting the pericardium. The main type detected was pericarditis, which was both macroscopically and microscopically confirmed. Pericarditis is commonly associated with hematogenous microbial infections, with a high prevalence of association with pneumonic lesions [24,25,48], which were detected in the animals analyzed. Non-suppurative myocarditis was described histologically in animals and could be associated with a wide variety of systemic diseases but is rarely primary [48]. The *Sarcocystis* spp. cysts reported histologically in the studied animals are a very common finding in ruminants and an increase in the numbers of cysts with age is confirmed [48].

Regarding the urinary system, interstitial nephritis was the most common histological finding, which was rarely detected grossly, which agrees with results of previous studies in goats [19,49].

## 5. Conclusions

The present study describes the most common concomitant pathologies found in goat herds with natural PTB infection mainly affecting the respiratory and gastrointestinal tracts. Our results emphasize the importance of routine histological examination for disease assessment, as well as the importance of anatomic pathology as a useful tool for the diagnosis and management of PTB in goat herds, since the majority of the described findings were only detectable histologically. Furthermore, vaccination against PTB might potentially have a positive effect on the reduction of respiratory and gastrointestinal pathologies, based on the findings described and the statistically significant differences demonstrated between the vaccinated and non-vaccinated animals. Further studies on a greater scale are needed to determine and understand the multiple etiologies and factors associated with the pathologies described, as well as the effect of PTB vaccination on their prevalence.

## Figures and Tables

**Table 1:** Number of vaccinated (V) and non-vaccinated (nV) goats included in the study by farm and age range.

Farm	Age Range (Months)						Total
	(0–12]	(12–24]	(24–36]	(36–48]	>48	Unknown	
F1	1V	2V	4V	-	-	1V	8V
F2	-	5V + 1nV	1V	-	-	-	6V + 1nV
F3	-	1V + 7nV	-	-	-	-	1V + 7nV
F4	-	5nV	1nV	-	-	-	6nV
F5	-	-	1nV	-	3nV	-	4nV
F6	-	-	2nV	-	-	-	2nV
F7	-	-	-	1nV	1nV	-	2nV
F8	-	-	1nV	1nV	-	-	2nV
Total (*n* = 39 ^1^)	1V	8V + 13nV	5V + 5nV	2nV	4nV	1V	15nV + 24nV

V, vaccinated; nV, non-vaccinated. ^1^ Data about the age of 38/39 animals was available. All animals included in the study presented histological lesions compatible with PTB, including granulomatous lymphadenitis of the mesenteric lymph nodes and/or granulomatous enteritis.

**Table 2:** Summary of MAP-induced morphological findings and lung pneumonic lesions in 39 naturally infected goats.

			Age Range (Months)								Total		
			(0–12]	(12–24]		(24–36]		(36–48]	>48	Unknown	V *n* = 15	nV *n* = 24	*n* = 39
Vaccination			V *n* = 1	V *n* = 8	nV *n* = 13	V *n* = 5	nV *n* = 5	nV *n* = 2	nV *n* = 4	V *n* = 1			
Gross PTB lesions	MS LNs	Yes	1 (100%)	6 (75%)	8 (61.5%)	4 (80%)	2 (40%)	1 (50%)	1 (25%)	1 (100%)	12 (80%)	12 (50%)	24 (61.5%)
		No	-	2 (25%)	5 (38.5%)	1 (20%)	3 (60%)	1 (50%)	3 (75%)	-	3 (20%)	12 (50%)	15 (38.5%)
	ICV	Yes	-	3 (37.5%)	1 (7.7%)	3 (60%)	-	-	-	-	6 (40%)	1 (4.2%)	7 (17.9%)
		No	1 (100%)	5 (62.5%)	12 (92.3%)	2 (40%)	5 (100%)	2 (100%)	4 (100%)	1 (100%)	9 (60%)	23 (95.4%)	32 (82.1%)
Histological PTB lesions	MS LNs (Severity grade)	I	-	-	3 (23.1%)	-	1(20%)	-	3 (75%)	-	-	7 (29.2%)	7 (17.9%)
		III	-	-	2 (15.4%)	-	-	-	-	-	-	2 (8.3%)	2 (5.1%)
		IV	1 (100%)	8 (100%)	7 (53.8%)	5 (100%)	3 (60%)	1 (50%)	1 (25%)	1 (100%)	15 (100%)	12 (50%)	27 (69.2%)
		Subtotal	1 (100%)	8 (100%)	12 (92.3%)	5 (100%)	4 (80%)	1 (50%)	4 (100%)	1 (100%)	15 (100%)	21 (87.5%)	36 (92.3%)
		No	-	-	1 (7.7%)	-	1 (20%)	1 (50%)	-	-	-	3 (12.5%)	3 (7.7%)
	ICV LP (severity)	Mild	-	5 (62.5%)	3 (23.1%)	4 (80%)	-	1 (50%)	1 (25%)	-	9 (60%)	5 (20.8%)	14 (35.9%)
		Moderate	-	-	1 (7.7%)	-	1 (20%)	1 (50%)	1 (25%)	-	-	4 (16.7%)	4 (10.3%)
		Marked	-	-	-	-	1 (20%)	-	1 (25%)	-	-	2 (8.3%)	2 (5.1%)
		Subtotal	-	5 (62.5%)	4 (30.8%)	4 (80%)	2 (40%)	2 (100%)	3 (75%)	-	9 (60%)	11 (45.8%)	20 (51.3%)
		No	1 (100%)	3 (37.5%)	9 (69.2%)	1 (20%)	3 (60%)	-	1 (25%)	1 (100%)	6 (40%)	13 (54.2%)	19 (48.7%)
Histological pneumonic lesions	Yes		-	7 (87.5%)	10 (76.9%)	-	4 (80%)	1 (50%)	2 (50%)	1 (100%)	8 (53.3%)	17 (70.8%)	25 (64.1%)
	No		1 (100%)	1 (12.5%)	3 (23.1%)	5 (100%)	1 (20%)	1 (50%)	2 (50%)	-	7 (46.7%)	7 (29.2%)	14 (35.9%)

PTB, paratuberculosis; MS LNs, mesenteric lymph nodes; ICV LP, ileocecal valve lamina propria; V, vaccinated; nV, non-vaccinated.

**Table 3:** Summary of Ziehl–Neelsen (ZN) and immunohistochemistry (IHC) results for MAP detection in 39 naturally infected goats.

			Age Range (Months)								Total		
			(0–12]	(12–24]		(24–36]		(36–48]	>48	Unknown	V *n* = 15	nV *n* = 24	*n* = 39
Vaccination			V *n* = 1	V *n* = 8	nV *n* = 13	V *n* = 5	nV *n* = 5	nV *n* = 2	nV *n* = 4	V *n* = 1			
MS LN	ZN	(+)	1 (100%)	6 (75%)	11 (84.4%)	4 (80%)	4 (80%)	2 (100%)	3 (75%)	1 (100%)	12 (80%)	20 (83.3%)	32 (82.1%)
		(−)	-	2 (35%)	2 (15.4%)	1 (20%)	1 (20%)	-	1 (25%)	-	3 (20%)	4 (16.7%)	7 (17.9%)
	IHC	(+)	1 (100%)	7 (87.5%)	10 (76.9%)	5 (100%)	4 (80%)	1 (50%)	4 (100%)	1 (100%)	14 (93.3%)	19 (79.2%)	33 (84.6%)
		(−)	-	1 (12.5%)	3 (23.1%)	-	1 (20%)	1 (50%)	-	-	1 (6.7%)	5 (20.8%)	6 (15.4%)
ICV LP	ZN	(+)	-	4 (50%)	7 (53.8%)	3 (60%)	3 (60%)	-	4 (100%)	-	7 (46.7%)	14 (58.3%)	21 (53.9%)
		(−)	1 (100%)	4 (50%)	6 (46.2%)	2 (40%)	2 (40%)	2 (100%)	-	1 (100%)	8 (53.3%)	10 (41.7%)	18 (46.1%)
	IHC	(+)	1 (100%)	5 (62.5%)	5 (38.5%)	3 (60%)	4 (80%)	1 (50%)	4 (100%)	-	9 (60%)	14 (58.3%)	23 (59%)
		(−)	-	3 (37.5%)	8 (61.5%)	2 (40%)	1 (20%)	1 (50%)	-	1 (100%)	6 (40%)	10 (41.7%)	16 (41%)

MS LN, mesenteric lymph node; ICV LP, ileocecal valve lamina propria; ZN, Ziehl–Neelsen; IHC, immunohistochemistry; (+) positive; (−) negative; V, vaccinated; nV, non-vaccinated.

## Data Availability

The data presented in this study are available on request from the corresponding author. The data are not publicly available due to privacy.

## Appendix A

**Table A1:** Summary of pathological findings in 39 naturally infected goats with PTB lesions.

System	Organ	Gross	Frequency (%)	Histology	Frequency (%)
Hemolymphatic	MS	LAM	21 (53.8%)	GLA	36 (92.3%)
		GLA	24 (61.5%)		
	RPh	GLA	2 (5.1%)	SLA/CLA	8 (20.5%)
		CLA	2 (5.1%)		
		Abscess	3 (7.7%)		
	PE	SLA	6 (15.4%)	SLA/CLA	4 (10.3%)
		Abscess	4 (10.3%)		
	MD	SLA	3 (7.7%)	SLA/CLA	8 (20.5%)
		CLA	3 (7.7%)		
		Abscess	2 (5.1%)		
	MA	SLA	1 (2.6%)	SLA/CLA	4 (10.3%)
		GLA CLA	2 (5.1%) 2 (5.1%)		
		Abscess	1 (2.6%)		
Respiratory	Lungs	CBP	4 (10.3%)	FPBP/FNBP	12 (30.8%)
		CBP + fibrinous pleuritis	7 (17.9%)	IP	6 (15.4%)
		CBP + fibrous pleuritis	5 (12.8%)	BIP	3 (7.7%)
				Pleuritis only	4 (10.3%)
Gastrointestinal	Intestine	Diffusely thickened appearance	10 (25.6%)	EE LPE	2 (5.1%) 1 (2.6%)
	Abomasum	PTB-compatible enteritis Hemorrhagic enteritis	7 (17.9%) 1 (2.6%)	GE	32(82.1%)
				Abomasitis/ abomasal ulcer	4 (10.3%)
	Liver	Steatosis	7 (17.9%)	Steatosis	11 (28.2%)
		Capsular fibrosis	6 (15.4%)	Lymphohistiocytic hepatitis	25 (64.1%)
		Mineralizations	1 (2.6%)	Granulomatous hepatitis	3 (7.7%)
Urinary	Kidney	Interstitial nephritis	2 (5.1%)	Interstitial nephritis	10 (25.6%)
Cardiovascular	Heart	Hydropericardium	2 (5.1%)	Non-suppurative myocarditis	3 (7.7%)
		Fibrous pericarditis	2 (5.1%)	Fibrous pericarditis	3 (7.7%)
		Petechial hemorrhages	2 (5.1%)	Myocardial sarcocystosis	9 (23.1%)

MS, mesenteric lymph nodes; RPh, retropharyngeal lymph nodes; PE, prescapular lymph nodes; MD, mediastinal lymph nodes; MA, mammary lymph nodes; LAM, lymphadenomegaly; GLA, granulomatous lymphadenitis; SLA, suppurative lymphadenitis; CLA, caseous lymphadenitis; CBP, cranioventral bronchopneumonia; FNBP, fibrinonecrotic brochopneumonia; FPBP, fibrinopurulent bronchopneumonia; IP, interstitial pneumonia; BIP, bronchointerstitial pneumonia; GE, granulomatous enteritis; EE, eosinophilic enteritis; LPE, lymphoplasmacytic enteritis.
